# Spirituality in Advanced Chronic Illness: The Lived Experience of Portuguese Patient–Caregiver Dyads

**DOI:** 10.3390/healthcare14203411

**Published:** 2026-10-13

**Authors:** Ana Eduarda Guiné, Letícia Figueirinha Crespo, Sidarth Pernencar, Ana Querido, Carlos Laranjeira

**Affiliations:** 1 School of Health Sciences, University of Leiria and Oeste, Rua General Norton de Matos, 2411-901 Leiria, Portugal; a.eduardaguine@hotmail.com (A.E.G.);; 2 Cardiology Department, Local Health Unit of the Leiria Region, Hospital of Santo André, Rua das Olhalvas, 2410-197 Leiria, Portugal; 3 Centre for Innovative Care and Health Technology (ciTechCare), Campus 5, Rua das Olhalvas, 2414-016 Leiria, Portugal; 4 RISE Health Research Group, University of Porto, 4200-450 Porto, Portugal; 5 Comprehensive Health Research Centre (CHRC), University of Évora, 7000-801 Évora, Portugal

**Keywords:** spirituality, caregivers, chronic disease, palliative care, qualitative research

## Abstract

**Background:** Spirituality is recognized as an essential dimension of palliative care, contributing to adaptation to suffering, vulnerability, and disease progression. Research has predominantly focused on either patients or caregivers separately, while the understanding of spirituality from a dyadic perspective remains limited. This study aimed to understand the lived experience of spirituality in the everyday lives of dyads composed of people living with advanced chronic illness and their informal caregivers. **Methods:** A qualitative study based on Colaizzi’s descriptive phenomenology was conducted. Thirteen dyads (n = 26 participants), comprising individuals with advanced chronic illness and their informal caregivers, were recruited from a Local Health Unit in the Central Region of Portugal using purposive sampling. Data were collected through semi-structured interviews and analysed according to Colaizzi’s seven-step method. The study was reported in accordance with the SRQR checklist. **Results:** Three main themes emerged from the analysis: (1) Spiritual Struggle and Challenges to Faith, characterized by disbelief, spiritual struggle, fragile hope, and divergent faith experiences; (2) Between Fate, Uncertainty, and Personal Agency, reflecting different ways of making sense of illness through fatalism, acceptance of life’s unpredictability, and a sense of personal control; and (3) Sources of Meaning and Spiritual Well-Being, encompassing family, nature and silence, life review, and prayer as resources that promoted comfort, hope, and meaning. **Conclusions:** Spirituality emerged as a dynamic, relational, and multifaceted experience that shaped how dyads interpreted, coped with, and attributed meaning to advanced illness. The findings highlight the need for palliative care approaches that systematically incorporate the spiritual dimension of both patients and caregivers as a unit of care.

## 1. Introduction

Population ageing and the increasing prevalence of non-communicable diseases are contributing to a substantial rise in the global need for palliative care (PC) [1]. Worldwide, the annual number of deaths is projected to increase from approximately 57 million in 2016 to more than 100 million by 2060, largely driven by chronic and progressive illnesses [2]. This demographic and epidemiological transition is expected to generate an unprecedented demand for PC services and support for individuals living with serious health-related suffering. In response to these needs, PC has been recognised as an essential component of healthcare. Radbruch et al. [3] define PC as an active and holistic approach aimed at improving the quality of life of individuals experiencing serious health-related suffering due to severe illness, as well as that of their families and caregivers. It seeks to prevent and alleviate suffering through the comprehensive assessment and management of physical, psychological, social, and spiritual concerns, while supporting patients throughout the illness trajectory and at the end of life. Despite its recognition as an essential component of healthcare, access remains limited: an estimated 56.8 million people worldwide require PC annually, yet fewer than 15% receive support from specialist services [4]. Importantly, the burden of advanced chronic illness extends beyond patients themselves, profoundly affecting family members and informal caregivers, who often play a central role in care provision and support throughout the illness trajectory [5].

Given the central role of caregivers in the illness experience, increasing attention has been directed toward understanding advanced chronic illness as a shared and relational process. Growing interest has emerged in understanding advanced chronic illness from a dyadic perspective, recognizing patients and family caregivers as interdependent members of the same relational unit [6]. This perspective is particularly relevant because the experience of living with advanced chronic illness extends far beyond its clinical manifestations, involving profound changes in life plans, autonomy, family roles, and perceptions of the future [7,8]. As the illness progresses, patients and caregivers often face shared physical, emotional, social, and existential challenges that shape and influence one another over time, requiring continuous adaptation from both members of the dyad [5,9]. In this context, how patients and caregivers attribute meaning to the illness experience is central to their psychological and existential adjustment [10,11]. Evidence highlighting the complexity and dynamic nature of these shared experiences underscores the need for a deeper understanding of dyads’ lived experiences [9].

Among the resources that may facilitate this process of adaptation and meaning-making, spirituality has attracted growing attention in PC research and practice. Spirituality has been identified as an important resource in this adaptation process, enabling individuals to cope with suffering, sustain hope, and find meaning in the face of adversity [12,13]. Understood as a multidimensional aspect of human existence, spirituality relates to the search for meaning, purpose, and transcendence and may be expressed through religion, nature, art, interpersonal relationships, or other forms of connection with oneself and others [14]. The literature suggests that spirituality can help alleviate the existential suffering frequently associated with advanced illness, promoting well-being and quality of life for both patients and caregivers [15,16]. Consequently, PC recognizes spiritual care as an essential component of a holistic, person- and family-centred approach.

The importance of spirituality may be particularly evident in advanced chronic conditions characterized by high symptom burden and substantial psychosocial and existential challenges. Among advanced chronic illnesses with a high need for PC, Chronic Obstructive Pulmonary Disease (COPD) and Heart Failure (HF) are particularly prominent. Although clinically distinct, both conditions are characterized by progressive trajectories, high symptom burden, significant functional impairment, and substantial impacts on quality of life [17]. These conditions affect not only patients but also their caregivers, who face increasing demands associated with caregiving responsibilities and uncertainty regarding disease progression.

In advanced COPD, alternating periods of stability and acute exacerbations, coupled with prognostic unpredictability, may intensify emotional distress for both patients and caregivers. Rubio et al. [18] demonstrated that the emotional suffering experienced by both members of the dyad is strongly interrelated, with reciprocal influences between the distress levels of patient and caregiver. The authors also highlighted the importance of interventions aimed at reducing caregiver burden, given its impact on the well-being of both members of the dyad. Moreover, evidence suggests that spiritual needs tend to increase as COPD progresses. Individuals in more advanced stages of the disease report lower levels of spiritual well-being and higher levels of anxiety, fear, and depression [19]. In this context, opportunities to express spiritual and existential concerns become particularly important, reinforcing recommendations for the progressive integration of spiritual care into the support provided to these individuals and their families [20].

Similarly, advanced HF is a complex condition that profoundly affects the lives of both patients and caregivers. A longitudinal study involving dyads of patients with advanced HF and their caregivers demonstrated that each member’s health status and psychological distress influence prognostic understanding and adaptation to illness, highlighting the interdependent nature of this experience [21]. Beyond physical and functional challenges, HF progression may trigger emotional, social, existential, and spiritual concerns, reinforcing the need for an integrated approach to care [22,23]. Nevertheless, the available evidence regarding spirituality in HF remains limited and fragmented, indicating a need for a deeper understanding of the spiritual needs and experiences of these individuals [24,25].

Taken together, these findings suggest that spirituality may play a significant role in helping patients and caregivers navigate the challenges associated with advanced COPD and HF. However, important gaps remain in understanding how these experiences are constructed and negotiated within the dyadic relationship. Despite the growing recognition of spirituality as an important resource for coping with advanced chronic illness [26], a significant gap remains in the literature regarding the understanding of this experience from a dyadic perspective. Research has largely focused on either patients or caregivers in isolation [27,28], leaving the relational and interdependent nature of spiritual experiences in advanced illness relatively unexplored. This limitation is particularly relevant in Portugal, where research on spirituality in advanced illness remains limited, especially studies examining patient and caregiver experiences from a dyadic perspective. Considering that advanced illness is often experienced as a shared process in which needs, meanings, and adaptive strategies mutually influence one another, there is a clear need to deepen understanding of how spirituality is experienced and constructed within this relationship.

To address this gap, the present study aimed to understand the lived experience of spirituality in the everyday lives of dyads composed of people living with advanced chronic illness and their informal caregivers. Specifically, the study sought to explore the essence of spirituality as experienced in the daily lives of dyads facing advanced chronic illness. To achieve this aim, the following research question was formulated: What is the essence of the lived experience of spirituality in the everyday lives of dyads in the context of advanced chronic illness? It is hoped that this study will contribute to understanding the spiritual dimension of the shared illness experience and support the development of more holistic, dyad-centred PC approaches.

## 2. Materials and Methods

### 2.1. Study Design

This study is part of a broader research project examining the experiences of people with advanced chronic illness and their informal caregivers. The same 13 dyads participated in another qualitative study derived from this project [29]; however, that investigation addressed different research questions and employed separate interview guides, interviews, and datasets.

The present study specifically focused on the lived experience of spirituality within patient–caregiver dyads and adopted a qualitative design grounded in Colaizzi’s descriptive phenomenological approach [30,31]. Grounded in Husserlian phenomenology, this approach seeks to describe the essence of a phenomenon as it is experienced by participants. Epistemologically, it assumes that knowledge emerges from the rigorous description of lived experiences and the meanings attributed to them, requiring researchers to adopt a phenomenological attitude and suspend their preconceptions through bracketing [32]. Given the subjective, relational, and contextual nature of spirituality, as well as the limited understanding of this phenomenon from a dyadic perspective, Colaizzi’s descriptive phenomenology was considered an appropriate approach for exploring how spirituality is experienced and understood in everyday life with advanced chronic illness.

This study was reported in accordance with the Standards for Reporting Qualitative Research (SRQR) (Table S1).

### 2.2. Setting and Participants

The study was conducted in a Local Health Unit located in the Central Region of Portugal and included participants receiving follow-up care in specialised Pulmonology and Advanced HP outpatient clinics. The institution provides healthcare services to a population of approximately 400,000 inhabitants, encompassing primary, hospital, and long-term care, and delivering specialised services for individuals with complex chronic illnesses and PC needs.

Participants were recruited using a purposive sampling strategy based on the principle of maximum variation, aiming to include individuals with diverse sociodemographic and clinical characteristics and, consequently, to maximise the range of experiences and perspectives related to the phenomenon under study.

Inclusion criteria for patients were (a) adults with a diagnosis of advanced COPD [classified as GOLD grade III (severe airflow limitation) or GOLD grade IV (very severe airflow limitation) [33] or advanced HF [defined as New York Heart Association (NYHA) functional class III or IV, indicating marked limitation of physical activity or symptoms at rest despite optimal medical management [34]; (b) ability to participate in an interview; and (c) identification of a significant informal caregiver willing to participate in the study. Inclusion criteria for informal caregivers were (a) age 18 years or older; (b) being identified by the patient as the primary informal caregiver; and (c) willingness to participate in the interview.

Participants in both groups were excluded if they presented cognitive impairment likely to compromise their understanding of the study or their ability to participate in an interview, or if they had uncontrolled symptoms that made data collection unfeasible at the time of recruitment.

Although participants were recruited as patient–primary caregiver dyads, the study did not assume that caregiving occurred exclusively within this relationship. Participants may also have received support from other family members, friends, community resources, or healthcare professionals. The dyad was adopted as the unit of analysis to explore the interdependent experiences of patients and their primary caregivers.

Sample size was guided by the principles of informational sufficiency and depth of experiential data commonly adopted in phenomenological research [31]. Recruitment continued until the research team determined that additional interviews were yielding limited novel insights regarding participants’ spiritual experiences and that sufficient richness had been obtained to support comprehensive thematic development. A total of 13 patient–caregiver dyads participated in the study, comprising individuals living with advanced chronic illness and their respective informal caregivers.

### 2.3. Data Collection

Data collection took place between November 2025 and February 2026. Potential participants were initially identified by healthcare professionals working in the Pulmonology and Cardiology outpatient clinics. Following their expression of interest and permission to be contacted, the researchers telephoned the dyads to explain the study objectives, answer any questions, and arrange an interview.

Interviews were conducted by the principal investigator (A.E.G.), a female social worker with prior training in qualitative research methodology and interviewing techniques. The interviewer had no prior relationship with the participants and was not involved in their clinical care. Data were collected through semi-structured interviews, a method that combines guiding questions with sufficient flexibility to explore emerging aspects of participants’ experiences. Before data collection commenced, a peer debriefing session was conducted with two experts in qualitative research (C.L. and A.Q.) to assess the clarity, relevance, and adequacy of the interview guide. No substantial modifications were considered necessary.

The interview guide was developed based on the available literature [35,36] and the researchers’ experience. It comprised two sections: the first focused on participants’ sociodemographic and clinical characteristics (age, gender, diagnosis, duration of illness, and relationship between dyad members), while the second explored experiences of spirituality. The main topics addressed included: (a) the meaning of spirituality in the context of advanced chronic illness; (b) spiritual resources used in everyday life; (c) the influence of illness on spirituality; and (d) the impact of spirituality on the patient–caregiver relationship. Examples of interview questions included: *“What does spirituality mean at this stage of your lives?”* *“How has spirituality helped you cope with the illness?”*, and *“How is spirituality experienced within your patient–caregiver relationship?”* When appropriate, probing questions were used to encourage reflection and further explore relevant issues emerging from participants’ narratives (e.g., *“How did you feel in that situation?”*, *“Could you describe that experience in more detail?”*, *“What exactly happened?”*).

All interviews were conducted face-to-face with both members of the dyad simultaneously, in a location chosen by the participants according to their preferences. This approach was intended to provide a comfortable environment that facilitated the expression of lived experiences. Prior to each interview, the researcher introduced herself, explained the study objectives, reinforced the principles of confidentiality and anonymity, and obtained written and verbal informed consent from all participants. Interviews were audio-recorded with participants’ permission and supplemented by field notes recorded in a reflexive journal following each interview. Interview duration ranged from approximately 45 to 90 min. Audio recordings were transcribed verbatim and imported into WebQDA software (Version 3.0, University of Aveiro, Aveiro, Portugal) for data management and organisation.

For the purposes of the present study, each participating dyad completed a single interview focused on spirituality and spiritual experiences. Transcripts were not returned to participants for verification due to their clinical vulnerability and the potential physical and emotional burden associated with living with advanced illness. Although participant validation is traditionally included as the final step of Colaizzi’s method [30], this procedure was not undertaken to minimise participant burden and avoid additional demands that could compromise their well-being. To ensure methodological rigor and trustworthiness, several alternative strategies were employed, including independent coding by two researchers, reflexive discussions, maintenance of field notes, bracketing procedures, and consensus meetings among the research team. All interviews were conducted in European Portuguese. Interview transcripts and the quotations presented in this manuscript were translated into English by the research team. To ensure linguistic accuracy and preservation of meaning, translations were independently reviewed by a bilingual researcher fluent in Portuguese and English, and discrepancies were discussed until consensus was reached.

### 2.4. Data Analysis

Data analysis followed Colaizzi’s seven-step method [30,31] and was informed by a dyadic perspective. Initially, all interviews were read repeatedly to gain a comprehensive understanding of participants’ experiences, considering both individual accounts and the dyad as an interdependent relational unit. Significant statements related to the experience of spirituality in the context of advanced illness were then identified from patient and caregiver narratives. Meanings were subsequently formulated from these statements, remaining grounded in participants’ descriptions while capturing how spirituality was experienced, interpreted, and negotiated within the dyadic relationship.

These formulated meanings were organized into thematic clusters based on conceptual similarities and relationships. Analysis proceeded at both individual and dyadic levels. Patient and caregiver narratives were first analysed separately and then systematically compared within each dyad to identify areas of convergence, divergence, complementarity, and mutual influence regarding spiritual beliefs, experiences, challenges, and sources of meaning. This iterative comparative process facilitated an in-depth understanding of spirituality as a relational phenomenon emerging within the shared context of advanced chronic illness, rather than solely as an individual experience.

The resulting thematic clusters were integrated into an exhaustive description of the phenomenon, reflecting the multiple dimensions of spirituality expressed by patients and caregivers. This description was subsequently refined into a fundamental structure capturing the essence of spirituality within the context of advanced illness and caregiving. Finally, the findings were synthesized into a comprehensive account of the lived experience of spirituality among dyads comprising individuals with advanced chronic illness and their informal caregivers.

To enhance analytical rigor, the analysis was conducted independently by two researchers (E.G. and C.L.). Formulated meanings, thematic clusters, and interpretations were subsequently compared and discussed until consensus was reached, thereby strengthening the credibility, dependability, and trustworthiness of the findings.

### 2.5. Rigour

The rigour of the study was ensured in accordance with the criteria of credibility, dependability, confirmability, and transferability proposed within Guba and Lincoln’s trustworthiness framework [37]. Credibility was enhanced through in-depth interviews, prolonged engagement with the data through repeated readings of the transcripts, and ongoing discussions among the researchers regarding emerging meanings throughout the analytical process. The credibility of the findings was further strengthened through investigator triangulation, involving the joint discussion and validation of emerging interpretations.

Throughout the study, the research team continuously reflected on the influence of their clinical, academic, and research experiences on data collection and interpretation, promoting a critical and rigorous analysis of the phenomenon under investigation. The team comprised a cardiologist (S.P.), two faculty members with expertise in qualitative research and PC (C.L. and A.Q.), and two social workers with advanced training in the field (A.E.G. and L.F.C.). This disciplinary diversity facilitated the triangulation of perspectives and contributed to a more comprehensive understanding of the phenomenon. Dependability was ensured through a detailed description of all methodological procedures, from participant recruitment to data analysis, allowing for the auditability and traceability of the research process. Confirmability was strengthened through the adoption of a phenomenological attitude during data collection and analysis, seeking to suspend preconceptions and prior assumptions (*bracketing*), as well as through the use of field notes and reflexive records documenting methodological decisions throughout the study. Transferability was supported by the detailed description of the study setting, participants, and data collection procedures, enabling readers to assess the applicability of the findings to similar contexts. In addition, the analysis rigorously followed the steps of Colaizzi’s descriptive phenomenological method, ensuring a rich and faithful description of the essence of the lived experience of spirituality among dyads composed of individuals living with advanced chronic illness and their informal caregivers.

### 2.6. Ethics

The study received approval from the Research Ethics Committee of the Local Health Unit of the Leiria Region (Approval No. 86/CEULSRL/2025). All procedures were conducted in accordance with the ethical principles of the Declaration of Helsinki and relevant data protection regulations. Prior to participation, all individuals received detailed verbal and written information regarding the study aims and procedures and provided written informed consent. Participation was entirely voluntary, and participants were assured that they could withdraw from the study at any stage without affecting the healthcare services they received. Audio recordings and verbatim transcripts were securely stored on password-protected digital devices accessible only to members of the research team. No financial or material incentives were offered for participation. To protect confidentiality and anonymity, participants were assigned alphanumeric identifiers. Quotations are attributed using codes that distinguish the dyad (e.g., D1, D2, D3) and, where relevant, the participant’s role within the dyad (e.g., Patient-D1; Caregiver-D1).

## 3. Results

### 3.1. Background of Participants

The sample comprised 13 patient–caregiver dyads (Table 1). A predominance of male patients was observed (69.2%, n = 9), while most caregivers were female. The majority of the dyads consisted of spouses, with only two comprising a patient and their daughter as the primary caregiver. Patients had a mean age of 73 years (range: 48–82 years), whereas caregivers had a mean age of 66 years (range: 41–80 years).

### 3.2. Interview Findings

**Figure 1:**
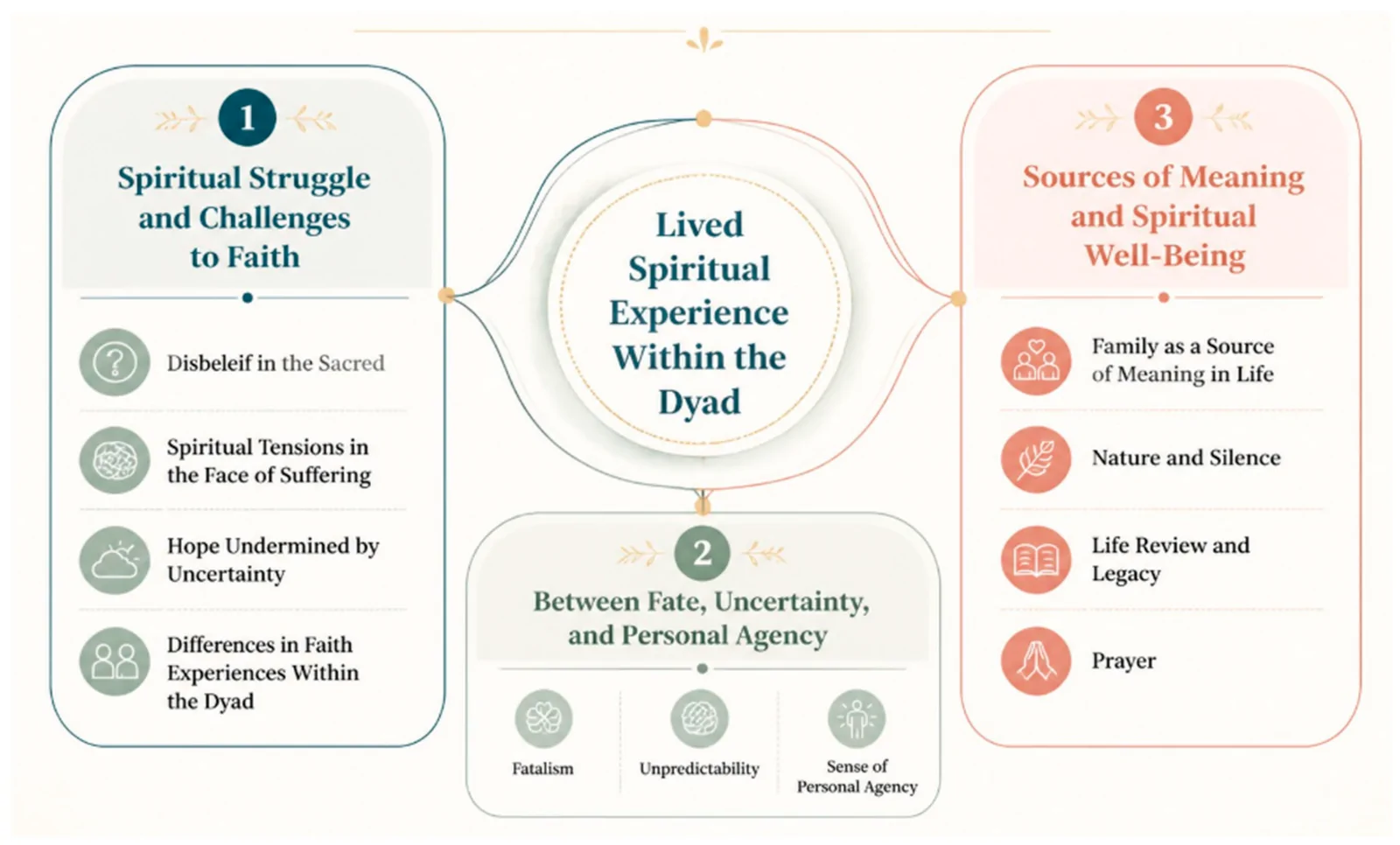
Representation of lived experience of spirituality in the everyday lives of dyads in the context of advanced chronic illness.

The analysis of the interviews provided insight into how dyads experience spirituality in the context of advanced chronic illness and caregiving, leading to the identification of three main themes (Figure 1). The themes identified were: (1) “Spiritual Struggle and Challenges to Faith,” reflecting experiences of spiritual questioning and the weakening of previously held beliefs; (2) “Between Fate, Uncertainty, and Personal Agency,” capturing different ways of attributing meaning to illness and life events; and (3) “Sources of Meaning and Spiritual Well-Being,” highlighting the resources that sustain comfort, hope, and adaptation to the illness experience. The findings are presented through illustrative excerpts from the interviews, accompanied by their respective phenomenological interpretations.

#### 3.2.1. Theme 1: Spiritual Struggle and Challenges to Faith

Disease progression, vulnerability, and an awareness of mortality led some dyads to profoundly reconfigure their spirituality. For these participants, the experience of illness did not necessarily strengthen their relationship with the sacred; instead, it triggered processes of questioning, distancing, and doubt regarding previously held beliefs. Spirituality therefore emerged as a space of tension between the need to find meaning and the difficulty in understanding the suffering they were experiencing. This theme comprises four subthemes: (a) Disbelief in the Sacred; (b) Spiritual Tensions in the Face of Suffering; (c) Hope Undermined by Uncertainty; and (d) Differences in Faith Experiences Within the Dyad.

(a) **Disbelief in the Sacred**

This subtheme reflects the erosion of previously valued beliefs and the difficulty in maintaining a trusting relationship with God or with religious institutions. Faced with the experience of illness and events perceived as unjust, some participants began to question the foundations of faith and the legitimacy of religious structures. For these dyads, spirituality ceased to be a source of comfort and security, giving way to feelings of disbelief and detachment.

As one patient reflected: *“I have never found answers in religion. For many years, I believed that faith helped people overcome difficult times, but after everything we have been through, I still have not found those answers. I kept praying and asking for help, yet the illness continued to progress, and I felt left alone with my suffering.”* (Patient-D3).

Echoing this sense of spiritual struggle, the caregiver described feelings of anger and questioning God: *“God is cruel. It is hard to believe otherwise when we see everything that has happened. We have always been people of faith, but there comes a point when we start asking why.”* (Caregiver-D3).

These accounts reflect not only the presence of religious doubts but also a deeper rupture with the sacred, marked by feelings of abandonment and by the inability to perceive faith as a meaningful resource for coping with illness. The suffering experienced appeared to undermine trust in a benevolent transcendent entity, leading to a spiritual experience characterised by scepticism, disillusionment, and detachment.

(b) **Spiritual Tensions in the Face of Suffering**

Anger and distress in response to suffering emerged as an expression of the conflict between the belief in a benevolent God and the reality of human suffering. Rather than representing a rejection of faith, this experience reflected an ongoing search for meaning in the face of events perceived as unjust, incomprehensible, and morally difficult to accept.

One dyad questioned God’s presence and action in situations of suffering considered incompatible with the notion of divine justice: Patient: *“If there is a God watching over us, there are things that simply cannot be understood*”; Caregiver: “*Especially when we see innocent people suffer”*; Patient: “*What did that child do wrong not to be cured?”;* Caregiver: *“Those situations are very difficult to reconcile with the idea of divine justice*.” (D9).

In this account, suffering emerged as a challenge to previously held beliefs, creating tension between faith and the need to make sense of events experienced as profoundly unjust. Illness and the suffering of vulnerable individuals appeared to challenge the image of a protective and benevolent God.

Despite these feelings of anger and questioning, there was also an effort to preserve a degree of spiritual coherence: *“Sometimes anger arises... But God cannot help everyone... perhaps there are things we simply cannot understand.”* (Caregiver-D9).

This oscillation between questioning and attempts at acceptance reflects the ambivalence characteristic of this subtheme. Anger did not necessarily signify a loss of spirituality; rather, it revealed an inner struggle to reconcile deeply rooted beliefs with an existential reality marked by pain, loss, and finitude. Suffering thus became a challenge to previously established systems of meaning, compelling dyads to renegotiate their understanding of God, justice, and the very meaning of existence.

(c) **Hope Undermined by Uncertainty**

Hope was weakened by disease progression and by a growing awareness of the limits of human control over life, health, and the future. Some dyads described feelings of resignation, uncertainty, and existential vulnerability, acknowledging that neither personal efforts nor medical advances are capable of fully altering the course of illness.

One patient stated: *“But unfortunately, that is how life is. There is no control. Medicine may ease things a little (...) but, in the end, it cannot change everything.”* (Patient-D5).

In this account, hope is confronted with the inevitability of the human condition, giving rise to an awareness of the limited capacity to control life events and their outcomes. Similarly, another caregiver commented: *“With all these things that are happening, a person loses hope (...) this world is lost (...) at my age, I never thought I would witness the things I have seen happening in recent times.”* (Caregiver-D2).

This narrative suggests that discouragement extends beyond the individual illness experience, reflecting a broader loss of confidence in the future and in the world around them. For these dyads, the experience of illness appeared to constrain their ability to envision future possibilities and to find meanings capable of sustaining hope. Spirituality, traditionally associated with transcendence, trust, and purpose, became fragile in the face of an increasing awareness of mortality, giving way to feelings of powerlessness and existential uncertainty.

(d) **Differences in Faith Experiences Within the Dyad**

Although some dyads shared the same experience of illness and caregiving, they did not always share the same relationship with faith and spirituality. In some cases, differing positions regarding religious beliefs were observed, with a stronger commitment to faith and religious practice coexisting alongside attitudes marked by distance, doubt, or ambivalence. These differences highlight that spirituality is a unique experience, shaped throughout each individual’s life trajectory and capable of assuming distinct meanings even within a shared experience.

One patient expressed an ambivalent position toward faith, stating: *“I am neither very religious nor completely non-believing. There are things I believe in and others about which I have many doubts. My wife sees things differently; she lives her faith”* (Patient-D9). This account reflects a spiritual experience characterized by the coexistence of belief and doubt, in which faith remains present but is accompanied by questioning and uncertainty. Rather than representing a rejection of the sacred, this experience suggests a dynamic and non-linear spiritual relationship, contrasting with the caregiver’s more committed and faith-oriented perspective.

In another dyad, the patient described a gradual distancing from religious practices: *“I’m not really connected to that anymore. I used to be more involved, but not now. She still goes to church, still prays, and still believes. I respect that, but that’s her way.”* (Patient-D11). In contrast, the caregiver from the same dyad remarked: *“What I pray extra, you pray less. Someone has to pray for both of us.”* (Caregiver-D11).

These accounts illustrate different ways of experiencing spirituality in the face of adversity. While prayer and faith remained important sources of comfort and hope for some caregivers, some patients demonstrated greater distance or ambivalence toward religious beliefs. Such differences reveal that spiritual experience in the context of advanced illness is not necessarily shared in a homogeneous manner, reflecting diverse ways of seeking meaning and coping with vulnerability.

#### 3.2.2. Theme 2: Between Fate, Uncertainty, and Personal Agency

This theme reflects the ways in which dyads made sense of advanced illness and its implications for their lives. As participants confronted suffering, uncertainty, and the prospect of decline, they drew on spiritual beliefs and values to interpret their experiences and locate meaning within them. Their accounts revealed an ongoing negotiation between acceptance of circumstances beyond their control and the desire to retain influence over their lives and decisions. This dynamic was expressed through three interconnected subthemes: (a) Fatalism, (b) Unpredictability, and (c) Sense of Personal Agency, each illustrating different ways of understanding the relationship between meaning, uncertainty, and personal control.

(a) **Fatalism**

Fatalism emerged as a way of understanding illness as part of a predetermined life path over which individuals have limited influence. For these dyads, spirituality was expressed through the belief that life events possess an inherent meaning or purpose, fostering an attitude of acceptance toward lived circumstances.

This perspective is illustrated by one patient who stated: *“When we are born, our destiny is already written. Some things happen because they are meant to happen, and there is no point in constantly asking why.”* (Patient-D4).

Similarly, another caregiver remarked: *“Nothing happens by chance. Even bad things happen for a reason and, sooner or later, we come to realize that they had a purpose in our lives.”* (Caregiver-D7).

In these accounts, illness is interpreted as part of a life journey that transcends individual will. The notion of fate or purpose serves as a way of organizing the experience of suffering, enabling difficult events to be incorporated into a meaningful narrative and fostering acceptance of the lived reality.

(b) **Unpredictability**

The unpredictability of life was reflected in participants’ awareness of human vulnerability and the impossibility of fully anticipating or controlling the course of life and illness. Faced with the uncertainty associated with the progression of advanced disease, some dyads acknowledged the limits of human understanding and the difficulty of finding explanations for certain events.

One dyad jointly reflected on the unpredictability of life and the limits of human control: Caregiver: “Life is very complicated. *We try to understand many things, but there are situations for which there is no explanation*”; Patient: *“One day a friend of ours seemed perfectly well, and the next day he died. It makes us realize how quickly life can change”*; Caregiver: *“The more we reflect on what happens to us, the more we realize how little control we actually have.”* (D8).

This perception was echoed by another caregiver, who noted: *“There are things we cannot control or avoid. No matter how much we try to understand or prepare ourselves, in the end we can only accept what life brings.”* (Caregiver-D13).

These accounts reveal an understanding of spirituality associated with accepting uncertainty and the fragility of the human condition. Rather than seeking definitive explanations for illness or suffering, participants acknowledged the inevitability of certain events and found in acceptance a way of coping with the unpredictability of existence.

(c) **Sense of Personal Agency**

A sense of personal agency emerged as an important source of meaning and spiritual adaptation to the experience of illness. Despite the limitations imposed by their health condition, participants recognized their capacity to influence their life trajectory through the choices, attitudes, and decisions they continued to make in everyday life. In this context, spirituality was associated with preserving autonomy and personal responsibility in the face of illness-related challenges.

One patient expressed this perspective by stating: *“We create our own destiny. What we achieve or lose depends largely on the decisions we make throughout our lives.”* (Patient-D1).

Similarly, other narratives suggested that, even in the face of uncertainty associated with disease progression, the ability to find meaning, make decisions, and respond to adversity constituted an important spiritual resource. As one caregiver explained: *“Faith helps us understand that we cannot control everything, but it also makes us believe that we still have a role to play. We may not be able to change the illness, but we can decide how we want to face this stage of life.”* (Caregiver-D12).

Together, these narratives suggest a spirituality grounded in meaning-making, responsibility, and the conviction that individuals retain agency in how they confront illness.

#### 3.2.3. Theme 3: Sources of Meaning and Spiritual Well-Being

The analysis revealed that, despite the challenges associated with advanced illness, dyads identified several sources of meaning, comfort, and serenity that contributed to their spiritual well-being. Spirituality was not expressed exclusively through religion or formal faith, but also through interpersonal relationships, connection with nature, reflection on one’s life journey, and spiritual practices. These experiences fostered feelings of continuity, belonging, hope, and inner peace, helping dyads integrate the experience of illness into their existential journey. This theme comprises four subthemes: (a) Family as a Source of Meaning in Life; (b) Nature and Silence; (c) Life Review and Legacy; and (d) Prayer.

(a) **Family as a Source of Meaning in Life**

Family relationships emerged as one of the most important sources of meaning and spiritual well-being. More than a support network, family was perceived as the primary reason to keep going, persevere, and find purpose despite the limitations imposed by illness. Family bonds enabled dyads to preserve a sense of belonging and continuity, reinforcing what they considered truly important in their lives.

As one caregiver explained, family had become the primary focus of daily life and a powerful source of motivation: *“Nowadays, it is really all about family. Nothing else matters much anymore. They are what gets me out of bed every day.”* (Caregiver-D11).

Likewise, another patient emphasized the restorative role of family relationships in sustaining emotional well-being and a sense of purpose amid illness: *“It is in my family that I find my well-being. When I am with them, I feel calmer, I forget my problems for a while, and I feel that life is still worth living.”* (Patient-D13).

These accounts suggest that spirituality was expressed through human connection and a sense of belonging, providing meaning to the illness experience and sustaining emotional and existential well-being.

(b) **Nature and Silence**

Nature and moments of silence emerged as important sources of tranquillity, introspection, and spiritual connection. For some dyads, these spaces provided opportunities to temporarily distance themselves from the demands and concerns associated with illness, fostering reflection, serenity, and reconnection with themselves and with what they considered truly meaningful.

One participant described the value of these moments as follows: *“Silence helps a lot. It gives you space to think about life and organize your thoughts. When we sit outside listening to the birds and feeling the sun on our faces, there is a peace that is impossible to explain.”* (Patient-D12).

In this account, nature transcended its physical dimension and became a space for reflection and inner well-being. The experience of contemplating nature appeared to promote a sense of calm and harmony that helped participants cope with the vulnerability and uncertainties associated with illness.

Similarly, another dyad highlighted the comfort found in peaceful environments and contact with nature: Patient: “*Sometimes we do not need to say anything*”; Caregiver: *“Just sitting together in the garden in silence already makes us feel better”*; Patient: “*It feels as if a weight has been lifted”;* Caregiver: “*And we can forget our problems for a while*”. (D6).

This testimony suggests that shared silence and being in nature constitute experiences of spiritual significance, providing moments of connection, inner peace, and emotional relief. For these dyads, spirituality was expressed not only through religious beliefs or practices but also through contemplation, stillness, and connection with the natural world as a source of comfort and meaning in the context of advanced illness.

(c) **Life Review and Legacy**

Reflecting on one’s life journey and on what would remain after death emerged as an important source of spiritual well-being. Recalling experiences, achievements, relationships, and contributions enabled dyads to recognize value and meaning in their lived experiences, fostering feelings of integrity and continuity.

One patient described how revisiting past experiences, achievements, and family relationships reinforced a sense that life had been meaningful and worthwhile: *“Sometimes I think about my life, the things I have done, the difficulties I have overcome, and the family I have built. It makes me feel that my life has been worthwhile.”* (Patient-D11).

Similarly, one dyad emphasized the importance of passing on their legacy to future generations: Patient: *“We talked openly about this with our children”*; Caregiver: *“It was very important to feel that who we are does not end here”*; Patient: *“In some way, what we leave behind in others continues after we are gone”*; Caregiver: *“That gives us a sense of continuity and peace”* (D13).

These narratives suggest that spirituality is expressed through the need to create coherence in one’s life story and to recognize the symbolic continuity of one’s identity beyond finitude.

(d) **Prayer**

Prayer emerged as an important source of comfort, hope, and inner strength. For these dyads, prayer represented a space for connecting with themselves, with God, and with that which transcended the immediate experience of illness. This practice was frequently associated with feelings of tranquillity, protection, and emotional support.

One caregiver stated: *“When the rosary is broadcast on the radio, we always make a point of listening to it. Afterwards, we feel more accompanied and more at peace.”* (Caregiver-D10).

Similarly, a patient explained that prayer helped to alleviate worry and distress, providing “*the strength to keep going*” despite not changing the circumstances of the illness (Patient-D11).

For another caregiver, attending Mass, either in person or through televised broadcasts, fostered *“a profound sense of peace”* and helped them feel better prepared to face future challenges (Caregiver-D12).

In these accounts, prayer emerged not merely as a religious practice but as an experience of comfort, connection, and transcendence, contributing to spiritual well-being and helping participants adapt to the challenges of advanced illness.

## 4. Discussion

This study contributes to the literature by exploring spirituality from a dyadic perspective, recognizing the patient and informal caregiver as an interdependent relational unit. Although the importance of spirituality in adapting to advanced illness has been widely acknowledged [38,39], existing evidence remains largely focused on individual experiences. Botas et al. [40] identified only a limited number of studies that simultaneously examined patients and caregivers, highlighting an important gap in understanding the spiritual dimension of living with advanced illness within a dyadic context. The present study therefore advances knowledge by illuminating spirituality as a relational experience characterized by mutual influences, convergences, and divergences in how dyads make sense of advanced illness.

The findings revealed spirituality as a dynamic, multifaceted experience profoundly shaped by encounters with vulnerability, suffering, and finitude. This understanding is consistent with the definition proposed by the European Association for Palliative Care, which conceptualizes spirituality as a dimension related to the search for meaning, purpose, and transcendence, expressed through relationships, beliefs, values, and connection to what is perceived as significant [41]. The findings suggest that spirituality extends beyond a strictly religious framework, emerging as a continuous process of meaning-making and meaning reconstruction in the context of advanced illness.

The first theme, “Spiritual Struggle and Challenges to Faith,” demonstrated that advanced illness may not only foster spiritual growth but may also be a source of spiritual distress. Some dyads described experiences of disbelief, religious questioning, anger in response to the perceived injustice of suffering, and diminished hope. The coexistence of faith, doubt, hope, and scepticism indicates that spirituality does not develop in a linear manner and may instead be characterized by tensions and internal conflicts. These findings are consistent with studies describing advanced illness as a catalyst for spiritual reappraisal, in which previously stable beliefs are challenged or reformulated in response to experiences of suffering and mortality [42]. Furthermore, the differences identified between members of the same dyad reinforce the notion that spirituality is simultaneously an individual and relational experience, capable of assuming different meanings even within a shared illness trajectory.

The second theme, “Between Fate, Uncertainty, and Personal Agency,” revealed different ways of understanding illness, suffering, and the course of life. Some dyads interpreted illness through a fatalistic lens, viewing events as part of a predetermined purpose or destiny. This perspective appeared to facilitate the integration of illness into a coherent and meaningful life narrative, fostering acceptance of events perceived as unavoidable. These findings are supported by literature describing spiritual fatalism as a strategy through which individuals seek to attribute meaning to difficult or seemingly uncontrollable events [43,44]. At the same time, participants demonstrated a strong awareness of life’s unpredictability and the limits of human control. Recognizing the uncertainty and vulnerability associated with disease progression appeared to promote processes of existential acceptance, as previously described by Jeserich et al. [45]. In contrast, other dyads emphasized their capacity to act, make decisions, and attribute meaning to their experiences, valuing autonomy and personal responsibility. Taken together, these findings suggest that dyads draw upon different spiritual frameworks to understand illness, oscillating between acceptance of forces that transcend human control and affirmation of personal agency in responding to adversity.

The third theme, “Sources of Meaning and Spiritual Well-Being,” highlighted the resources that supported comfort, hope, and adaptation throughout the illness trajectory. Among these, family emerged as the primary source of meaning and purpose. Beyond providing practical support, family relationships represented a space of belonging, continuity, and emotional connection, supporting Puchalski et al.’s [15] model of spirituality, which identifies interpersonal relationships as a central domain of spiritual experience. Prayer, nature, and moments of silence were also identified as important sources of spiritual well-being. These resources promoted feelings of inner peace, tranquillity, and hope, helping dyads cope with the emotional and existential demands of advanced illness. Such findings are consistent with evidence describing prayer as a widely used spiritual coping strategy in the face of suffering and uncertainty [46], as well as studies recognizing nature and contemplative experiences as sources of existential connection and transcendence [47,48]. Life review and legacy building also emerged as important sources of meaning. Reflecting on memories, relationships, and contributions made to others enabled participants to preserve a sense of continuity and integrity despite an awareness of mortality. These findings converge with the principles of Dignity Therapy, which emphasize the importance of life narratives, legacy, and the transmission of values in promoting spiritual well-being among people living with advanced illness [49]. These findings are also consistent with the work of Timóteo et al. [50] and Laranjeira et al. [51], which emphasize the value of life narratives, meaning-making, and legacy as central dimensions of spirituality in contexts of vulnerability and finitude. The recognition of the impact left through relationships, family, and future generations appears to foster feelings of identity continuity, transcendence, and purpose, constituting an important resource for existential adaptation in the context of advanced illness [50,52].

Across all three themes, the findings demonstrated that spirituality is not experienced solely at the individual level but also within the shared relational space between patients and caregivers. The dyadic analysis revealed that meanings attributed to faith, suffering, hope, destiny, and sources of spiritual well-being are continuously negotiated and reconstructed within the relationship. This evidence reinforces the value of dyad-centred approaches in PC, acknowledging that spiritual needs and adaptation processes are often interconnected and mutually influential. Consequently, this study broadens current understanding of spirituality in advanced illness and supports the development of interventions that recognize both the patient and the caregiver as a unit of care.

One of the principal contributions of this study is its demonstration of the relational nature of spirituality within the context of advanced chronic illness. While spirituality was experienced and interpreted individually, participants’ accounts revealed that spiritual beliefs, values, and sources of meaning were frequently shaped within the dyadic relationship. Experiences of faith, suffering, uncertainty, hope, and meaning-making often reflected processes of mutual influence, in which the beliefs, concerns, and coping responses of one member informed and shaped those of the other. At the same time, differences in spiritual perspectives highlighted that shared life circumstances do not necessarily lead to shared interpretations or experiences. Together, these findings underscore spirituality as both a personal and relational phenomenon and highlight the value of adopting dyadic approaches to better understand spiritual experiences in PC.

An additional consideration relates to the conceptual breadth of spirituality adopted in this study. Consistent with contemporary palliative care frameworks, spirituality was understood as encompassing meaning, purpose, relationships, and connection to what individuals perceive as significant or transcendent [53,54]. However, several dimensions identified in participants’ narratives, particularly family relationships, connection with nature, and meaning-making processes, may overlap with related constructs such as psychological coping, resilience, social support, and broader humanistic values. As a result, distinguishing specifically spiritual experiences from other psychosocial resources is not always straightforward. In keeping with the phenomenological orientation of this study, priority was given to participants’ own understandings and interpretations of what they considered spiritually significant. Nevertheless, this potential conceptual overlap should be considered when interpreting the findings and points to the continuing need for greater conceptual clarity and refinement within spirituality research.

### 4.1. Strengths and Limitations of the Study

The main strength of this study lies in the adoption of a phenomenological approach, which proved particularly appropriate for exploring and understanding the meanings attributed to spirituality by individuals living with advanced illness and their caregivers. This methodology enabled access to the richness and depth of participants’ lived experiences, fostering a comprehensive understanding of the phenomenon from their own perspective. A further strength is the dyadic approach, which moved beyond a perspective focused exclusively on either the patient or the caregiver, allowing spirituality to be understood as a simultaneously individual, relational, and interdependent experience. Additionally, the use of semi-structured interviews provided the flexibility necessary to explore emerging themes and to deepen aspects considered meaningful by participants.

Despite these contributions, several limitations should be considered when interpreting the findings. First, participants were recruited from a single healthcare institution in the Central region of Portugal, which may limit the transferability of the findings to other sociocultural and organizational contexts. Although purposive sampling with maximum variation was used to ensure diversity of experiences, the findings reflect a specific care context and should be interpreted accordingly. Second, the recruitment of dyads proved particularly challenging due to the clinical vulnerability of some patients and the voluntary refusal of participation by potential participants. Furthermore, in some interviews, participants’ health status, fatigue, and exhaustion influenced the duration and depth of their narratives. Consequently, the possibility of selection bias cannot be excluded, as participating dyads may differ in relevant ways from those who were unable or unwilling to participate. Nevertheless, respecting participants’ autonomy and avoiding any form of pressure to participate was considered an ethically necessary decision, safeguarding both their well-being and the voluntary nature of participation.

It should also be noted that the cross-sectional design enabled the exploration of spirituality at a single point in the illness trajectory and did not allow for an understanding of how spiritual experiences may evolve over time or across different stages of illness. Longitudinal investigations may provide further insight into the ways spirituality changes as illness progresses. Finally, conducting interviews in a dyadic context may have limited the expression of more sensitive issues or experiences that participants preferred not to disclose in the presence of the other dyad member. Although this methodological choice enabled the exploration of relational dynamics associated with spirituality, it may also have influenced the depth or spontaneity of some individual narratives. Subsequent studies could therefore combine dyadic and individual interviews, enabling the simultaneous exploration of jointly constructed meanings and more private spiritual experiences. In addition, the present findings highlight the need for further empirical investigation of dyadic spiritual care interventions, including their potential impact on spiritual well-being, adaptation to illness, hope, and caregiver burden.

Another limitation relates to the study’s focus on patient–primary caregiver dyads. Although this approach enabled an in-depth exploration of interdependent experiences, it did not systematically assess the influence of broader caregiving networks, including extended family members, friends, community resources, or formal healthcare services. These external sources of support may shape both coping processes and spiritual experiences. Future studies could adopt wider relational perspectives to explore how spirituality is experienced and negotiated across broader care networks.

Finally, participant validation of transcripts and findings (member checking), traditionally considered the final step of Colaizzi’s method, was not undertaken. This decision was made to minimise the physical and emotional burden placed on participants living with advanced chronic illness. Consequently, participants did not have the opportunity to verify the accuracy of interpretations or provide additional reflections on the findings. Although the absence of member checking may limit the confirmability of the results, rigor was supported through investigator triangulation, reflexive journaling, bracketing procedures, peer debriefing, and regular discussions among the research team regarding emerging interpretations and thematic development.

### 4.2. Implications for Practice

The findings of this study have important implications for clinical practice, particularly in the fields of PC and advanced chronic illness management. First, they highlight the need for the systematic assessment of the spiritual needs of both patients and informal caregivers, recognizing that spirituality may function not only as a source of hope, meaning, and adaptation but also as a source of doubt, conflict, and existential distress. This assessment could be facilitated through the routine use of validated spiritual screening and assessment tools, enabling healthcare professionals to identify spiritual resources, unmet needs, and areas of suffering that may otherwise remain unaddressed [55]. Second, the findings reinforce the importance of adopting a dyad-centred approach, as understandings of illness, suffering, and spirituality frequently emerge within a relational context characterized by mutual influence. Spiritual care interventions should therefore not be limited to the patient alone but should also recognize the informal caregiver as an active participant in the illness experience [9].

The findings further demonstrate that beliefs related to destiny, life’s unpredictability, and personal agency influence how dyads cope with illness progression. Healthcare professionals should therefore be prepared to explore and accommodate different belief systems and meaning-making processes while respecting the uniqueness of each individual’s experience and avoiding assumptions about how spirituality is lived and expressed. Finally, the results suggest the need for healthcare professionals to receive training in spiritual care, including the use of validated spiritual assessment instruments, as well as in communication skills that facilitate discussions about existential concerns, hope, meaning, suffering, and purpose in life. Such competencies may contribute to the delivery of more holistic, individualized, and dyad-centred care for patients with advanced illness and their caregivers.

## 5. Conclusions

This study provided insights into spirituality as a dynamic, relational experience profoundly influenced by living with advanced chronic illness. From a dyadic perspective, the findings revealed that spirituality can serve simultaneously as a source of meaning, hope, comfort, and adaptation, while also representing a space for doubt, conflict, and existential questioning. Dyads drew upon different interpretive frameworks to understand illness and suffering, oscillating between acceptance of destiny, recognition of uncertainty, and the affirmation of personal agency in the face of adversity. The findings further demonstrated that spirituality extends beyond formal religiosity, being expressed through family relationships, connection with nature, moments of silence, life review, and prayer. These sources of meaning contributed to the preservation of spiritual well-being and supported adaptation to the challenges imposed by disease progression. By highlighting the interdependent nature of the spiritual experiences of patients and caregivers, this study reinforces the importance of dyad-centred approaches in PC and advanced illness. The systematic integration of spiritual assessment and support may contribute to more holistic, person-centred, and compassionate care that is responsive to the existential needs of both members of the dyad. Future research should explore how spirituality evolves across the illness trajectory and evaluate spiritual care interventions designed to support both patients and informal caregivers simultaneously.

## Figures and Tables

**Table 1:** Sociodemographic and clinical characteristics of patient–caregiver dyads.

Variables	Patients (n = 13)	Caregivers (n = 13)
**Sex**		
Male	9	4
Female	4	9
**Age [years] (mean ± SD)**	73.39 ± 9.47	65.77 ± 12.50
**Relation to patient**		
Husband/Wife	-	11
Daughter	-	2
**Diagnosis ¥**		
COPD (GOLD III and IV)	5	-
HF (NYHA III and IV)	8	-
**Time of Diagnosis (mean ± SD)**	6.58 ± 8.0	-

¥ GOLD [Global Initiative for Chronic Obstructive Lung Disease] grade III and GOLD grade IV; New York Heart Association (NYHA) functional class III and class IV.

## Data Availability

The data presented in this study are available on reasonable request from the corresponding author. The data are not publicly available due to privacy and ethical restrictions relating to the protection and confidentiality of human-participant data. This article is based on the first author’s master’s dissertation in Palliative Care at the School of Health Sciences—University of Leiria and Oeste.

## Supplementary Materials

The following supporting information can be downloaded at: https://www.mdpi.com/article/10.3390/healthcare14203411/s1, Table S1: Standards for Reporting Qualitative Research (SRQR) checklist. Ref. [56] is cited in the Supplementary Material file.

## Abbreviations

The following abbreviations are used in this manuscript:

COPD	Chronic Obstructive Pulmonary Disease
GOLD	Global Initiative for Chronic Obstructive Lung Disease
ESAS	Edmonton Symptom Assessment Scale
HF	Heart Failure
NYHA	New York Heart Association
PC	Palliative Care
