# Supplementary material for: Spirituality in Advanced Chronic Illness: The Lived Experience of Portuguese Patient–Caregiver Dyads
Source: Healthcare (Basel). 2026 Oct 13;14(20):3411. doi: 10.3390/healthcare14203411 (PMC13704280; doi:10.3390/healthcare14203411)
Supplement: Supplementary file 1 [file healthcare-14-03411-s001.zip › healthcare-4572230-supplementary.pdf]

Table S1: Standards for Reporting Qualitative Research (SRQR) checklist.
http://www.equator-network.org/reporting-guidelines/srqr/

Page/line no(s).

Title and abstract

**Title** - Concise description of the nature and topic of the study Identifying the study as qualitative or indicating the approach (e.g., ethnography, grounded theory) or data collection methods (e.g., interview, focus group) is recommended.	Pag. 1; lines 2-3
**Abstract** - Summary of key elements of the study using the abstract format of the intended publication; typically includes background, purpose, methods, results, and conclusions.	Pag. 1; lines 16-38

Introduction

**Problem formulation** - Description and significance of the problem/phenomenon studied; review of relevant theory and empirical work; problem statement.	Pag. 2-3; lines 43-134
**Purpose or research questio**n - Purpose of the study and specific objectives or questions.	Pag. 2; lines 135-143

Methods

**Qualitative approach and research paradigm** - Qualitative approach (e.g., ethnography, grounded theory, case study, phenomenology, narrative research) and guiding theory if appropriate; identifying the research paradigm (e.g., postpositivist, constructivist/ interpretivist) is also recommended.	Pag. 4; lines 147-162
**Researcher characteristics and reflexivity** - Researchers' characteristics that may influence the research, including personal attributes, qualifications/experience, relationship with participants, assumptions, and/or presuppositions; potential or actual interaction between researchers' characteristics and the research questions, approach, methods, results, and/or transferability.	Pag. 6-7; lines 291-297
**Context** - Setting/site and salient contextual factors.	Pag. 4; lines 165-171
**Sampling strategy** - How and why research participants, documents, or events were selected; criteria for deciding when no further sampling was necessary (e.g., sampling saturation).	Pag. 4; lines 172-188
**Ethical issues pertaining to human subjects** - Documentation of approval by an appropriate ethics review board and participant consent, or explanation for lack thereof; other confidentiality and data security issues.	Pag. 7; lines 310-324
**Data collection methods** - Types of data collected; details of data collection procedures including (as appropriate) start and stop dates of data collection and analysis, iterative process, triangulation of sources/methods, and modification of procedures in response to evolving study findings.	Pag. 4; lines 185-231
**Data collection instruments and technologies** - Description of instruments (e.g., interview guides, questionnaires) and devices (e.g., audio recorders) used for data collection; if/how the instrument(s) changed over the course of the study.	Pag. 5; lines 201-253
**Units of study** - Number and relevant characteristics of participants, documents, or events included in the study; level of participation (could be reported in results).	Pag. 7-8; lines 326-334
**Data processing** - Methods for processing data prior to and during analysis, including transcription, data entry, data management and security, verification of data integrity, data coding, and anonymization/de-identification of excerpts.	Pag. 6; lines 254-281

**Data analysis** - Process by which inferences, themes, etc., were identified and developed, including the researchers involved in data analysis; usually references a specific paradigm or approach.	Pag. 6; lines 254-281
**Techniques to enhance trustworthiness** - Techniques to enhance trustworthiness and credibility of data analysis (e.g., member checking, audit trail, triangulation).	Pag.6-7; lines 282-309

Results/findings

**Synthesis and interpretation** - Main findings (e.g., interpretations, inferences, and themes); might include development of a theory or model, or integration with prior research or theory	Pag. 8-13; lines: 335-594
**Links to empirical data** - Evidence (e.g., quotes, field notes, text excerpts, photographs) to substantiate analytic findings	Pag. 8-13; lines: 335-594

Discussion

**Integration with prior work, implications, transferability, and contribution(s) to the field -** Short summary of main findings; explanation of how findings and conclusions connect to, support, elaborate on, or challenge conclusions of earlier scholarship; discussion of scope of application/generalizability; identification of unique contribution(s) to scholarship in a discipline or field	Pag.13-15; lines 595-700
**Limitations** - Trustworthiness and limitations of findings	Pag. 15-16; lines 701-756

Other

**Conflicts of interest** - Potential sources of influence or perceived influence on study conduct and conclusions; how these were managed	Pag. 18; lines 823-825
**Funding** - Sources of funding and other support; role of funders in data collection, interpretation, and reporting	Pag. 18; lines 811-814

O'Brien BC, Harris IB, Beckman TJ, Reed DA, Cook DA. Standards for reporting qualitative research: a synthesis of recommendations. *Academic Medicine*, 2014, 89, 9. doi: 10.1097/ACM.0000000000000388
